# Use of a Cardiopulmonary Resuscitation Video to Assist Intensive Care Unit Resident Physicians during Code Status Discussions

**DOI:** 10.1089/pmr.2022.0006

**Published:** 2022-09-06

**Authors:** Trinh T. Pham, Israel Acosta Sanchez, Salil Kalra, Sarung Kashyap, June Mbae, Natalie Marie Punal, Maria Panlilio, Daren Heyland, Tirsa M. Ferrer Marrero

**Affiliations:** ^1^ Department of Medicine, Division of Pulmonary, Critical Care, Sleep and Allergy, University of Illinois at Chicago, Chicago, Illinois, USA.; ^2^ Department of Critical Care Medicine, School of Medicine, Queen's University, Kingston, Ontario, Canada.

**Keywords:** code status discussions, CPR video, intensive care unit, resident physicians

## Abstract

**Background:**

Code status discussions (CSDs) in the intensive care unit (ICU) are frequently conducted by resident physicians. Cardiopulmonary resuscitation (CPR) videos when used to aid ICU patients and families in code status decision making have been shown to have a positive impact. The purpose of this study is to evaluate the impact of a CPR video, when made available to supplement trainee–patient CSDs, on ICU residents' comfort level when conducting these discussions.

**Objectives:**

To assess whether a CPR video as an intervention tool would increase residents' comfort level when conducting CSDs.

**Methods:**

This is a pre- and postintervention pilot study. A presurvey querying details about trainees' comfort level when conducting CSDs was administered to the residents at the beginning of the ICU rotation, and a CPR video was availed to them to supplement their trainee–patient CSDs. A postsurvey was administered to trainees at the end of their ICU rotation to evaluate and analyze the impact of the CPR video on residents' comfort level when conducting trainee–patient CSDs.

**Results:**

A total of 118 trainees rotated through the ICU with 43 (36%) answering the presurvey and 28 (24%) answering the postsurvey. Twenty-two (51%) presurvey respondents felt extremely comfortable and 18 (42%) felt somewhat comfortable conducting CSDs. Thirteen (46%) postsurvey respondents felt extremely comfortable and 12 (43%) felt somewhat comfortable conducting CSDs. Most postsurvey respondents (79%) almost never used the video and (67%) neither agree nor disagree that the video was useful.

**Conclusion:**

In our small cohort, CPR video when made available to supplement trainee–patient CSDs did not impact resident physicians' comfort level when conducting these discussions. The residents' low level of engagement with this video, among other factors, could explain our results.

## Introduction

Code status discussions (CSDs) in the inpatient setting are frequently performed by resident physicians in training.^1^ CSDs are critical and sensitive conversations, especially in the intensive care unit (ICU), and can be challenging for residents to carry out effectively.^2^ However, although these conversations can be challenging, residents often assume the responsibility of CSDs as a standard part of their training and physician duty.^3,4^

Siddiqui and Holley^4^ published the results of a survey that was distributed to 18 internal medicine (IM) residency programs. Only one-third of the respondent residents felt “very comfortable” with CSDs.^4^ Ury et al.^5^ published similar findings, demonstrating that most incoming medical residents have low self-confidence when leading end-of-life conversations with patients and families. Mills et al.^6^ surveyed fourth-year medical students who were matched into an IM residency program. Most students reported having moderate levels of self-confidence in conducting CSDs independently.

However, their self-confidence decreased in particular domains, such as when describing the literature on cardiopulmonary resuscitation (CPR), predicting outcomes, or recommending a code status.^6^ These findings raised important questions regarding whether allocating further education and resources would help residents to confidently and comfortably facilitate CSDs or not.

Since ∼20% of deaths in the United States occur in the ICU,^7^ it is important for residents to feel comfortable engaging in effective communication during CSDs. This helps ensure that patients' goals of care are adequately addressed and can significantly impact their clinical course. Moreover, effective CSDs can help families and surrogates have a more trusting and open dialogue with the ICU team, thus, helping lessen the anxiety and depression they often experience.^7,8^

Communication in end-of-life–related topics is a core competency listed by the Accreditation Council of Graduate Medical Education for physicians in training. It is also assessed on the American Board of Internal Medicine certification examination.^7^ In addition, the Center for Medicare and Medicaid Services launched the Hospital Consumer Assessment of Health Care Providers and System survey in which patients answer questions about physician's communication. Interestingly, reimbursement is contingent upon documentation of this survey's data.^6,7^ Therefore, it is important for residents to feel comfortable conducting CSDs, as these conversations will assuredly impact their patients' care and their future career as physicians.

Several institutions have implemented curriculums utilizing multimodal training to improve residents' confidence in conducting CSDs.^9^ Often, these interventions include interactive presentations, live role play, and pre- and postintervention tests.^2^ Smith et al.^7^ implemented a curriculum with a pre- and postintervention questionnaire. In the postintervention questionnaire, the residents reported that the intervention increased their comfort level in CSDs and their confidence when answering end-of-life questions to patients and families.^7^

CPR videos have been shown to have a positive impact on ICU patients and families when deciding code status.^8^ To our knowledge, the effect of CPR videos on residents' comfort level when conducting CSDs has not been studied, especially when the video is provided for them to frame or supplement these discussions.

In this report, we discuss our experience piloting a pre- and postintervention study project in which a CPR video was offered to ICU rotating residents as an audiovisual tool to supplement their CSDs with patients.

Our objective is to assess the impact of a CPR video on the residents' level of comfort when conducting the CSD and their perception of the patients' understanding and satisfaction with the information provided.

## Methods

A pre- and postintervention pilot study was conducted at our university hospital ICU from February 1st to October 30th, 2021, in which a CPR video was offered to the ICU rotating residents as an audiovisual tool to supplement their CSDs with patients.

In a collaborative process, the authors of this project developed a pre- and postsurvey that were administered to all the ICU rotating residents at the beginning and end of their rotation, respectively. After a literature review, a list of questions was created as the first draft of the survey. The questions were further modified to ensure cogency. Both the pre- and postsurvey were reviewed by a pulmonary and critical care faculty physician not affiliated with the project for further face and content validity.

The survey links were sent to all categorical IM and preliminary residents over a secure institutional e-mail. Participation was anonymous and voluntary, and no incentives were offered for participation. The presurvey was a 12-item questionnaire with multiple choice questions querying demographics and the residents' perceived frequency of the CSDs they conduct in the ICU. The presurvey also included Likert items and scales regarding residents' level of comfort conducting CSDs, their perception of patients' overall understanding and satisfaction, and the content of these discussions.

The residents watched the English version of the CPR video at the beginning of their ICU rotation, and they were recommended to use it as a personal reference to frame their CSDs. Links to both the English and Spanish versions of the CPR video were made available to the residents through e-mail and on a smart tablet located in the ICU. The residents were encouraged to use the CPR video as a discussion tool they could show to patients and families while having the CSD.

The audiovisual tool is a seven-minute CPR video titled “A decision aid to prepare patients and their families for shared decision making about cardiopulmonary resuscitation (CPR).” Dr. Daren Heyland and colleagues from the Canadian Researchers at the End-of-Life Network developed the video in 2012. Permission was obtained from Dr. Heyland for the use of the CPR video as well as its translation into Spanish. The video starts with an introduction explaining cardiac arrest.^8^ It describes CPR with simplified illustrations and discusses the survival rate data. It also addresses the impact of the patient's prior comorbidities on the survival rate and the likelihood of neurological sequelae in survivors.^8^ The last section is a reflection on deciding on code status.^8^

After permission for the video translation was obtained, the content of the video was translated and recorded by a native Spanish-speaking health care professional with experience in both community and health care translation.^10^ The initial translation was literal, focusing on the accurate communication of the content. The translation was revised and attested by a second native Spanish-speaking health care professional to ensure accurate language reproduction within the contextual framework of the CPR video, with special attention to the colloquial terms and common cultural expressions often used by our hospitals' Spanish-speaking patient population.^10^

The postsurvey was a 15-item questionnaire with questions identical to the presurvey as already detailed with the addition of questions pertaining to the CPR video, querying whether the resident used the CPR video, in what capacity it was used, and whether the video was considered helpful.

The surveys were collected anonymously using the institutional cloud-based subscription to a software platform and were password secured within the Division of Pulmonary, Critical Care, Sleep and Allergy servers at the University of Illinois at Chicago, Department of Medicine.

The statistical methods used were descriptive statistics of both nominal and ordinal variables, using percentages to express the frequency distribution. For the primary outcome, which was the residents' reported comfort level when conducting CSDs, descriptive statistics were performed with percentages to express the frequency distribution, and median and mode to express central tendencies. Given the pilot nature of this study, no formal sample size or power calculation was performed.

This project was approved by the University of Illinois at Chicago Institutional Review Board/Office of the Protection of Research Subject and by the Internal Medicine Residency Program Director.

## Results

From February 1st to October 30th, 2021, a total of 118 trainees rotated through the ICU. A total of 43 residents answered the presurvey (36%, *N* = 43/118) and 28 (24%, *N* = 28/118) answered the postsurvey.

Of the presurvey respondents, the majority were male (72%, *N* = 31). Sixteen trainees (37%) were postgraduate year (PGY)1, 9 (21%) were PGY2, 16 (37%) were PGY3, and 2 (5%) trainees were other. The majority were IM categorical residents (58%, *N* = 25). Thirty-eight (88%) of the respondents answered they would discuss code status with 100% of the patients they would admit to ICU.

Twenty-two (51%) of the presurvey respondents answered feeling extremely comfortable conducting CSDs, 18 (42%) answered somewhat comfortable, 2 (5%) answered neither comfortable nor uncomfortable, and 1 (2%) answered feeling somewhat uncomfortable (Table 1). The response option answered with a higher frequency is “extremely comfortable” and the median is “somewhat comfortable.”

**Table 1.** Comparison of Pre- and Postintervention Survey Responses for the Primary and Secondary Outcomes

	Presurvey (***N*** = 43/118, 36%)	Postsurvey (***N*** = 28/118, 24%)
Primary outcome, *n* (%)		
Q1. How comfortable are you discussing code status with a patient on your own?		
Extremely comfortable	22 (51)	13 (46)
Somewhat comfortable	18 (42)	12 (43)
Neither comfortable nor uncomfortable	2 (5)	3 (11)
Somewhat uncomfortable	1 (2)	0 (0)
Extremely uncomfortable	0 (0)	0 (0)
Secondary outcome, *n* (%)		
Q1. After conducting code status discussions, how frequently do you feel confident the patient understood the information provided?		
Nearly always	6 (14)	3 (11)
Often	23 (53)	14 (50)
Sometimes	11 (26)	9 (32)
Occasionally	3 (7)	2 (7)
Almost never	0 (0)	0 (0)
Q2. After conducting a code status discussion, how frequently do you feel confident that the patient is satisfied with the information provided?		
Nearly always	3 (7)	3 (11)
Often	27 (63)	14 (50)
Sometimes	13 (30)	11 (39)
Occasionally	0 (0)	0 (0)
Almost never	0 (0)	0 (0)

Values shown represent subject numbers (*N*) and percent (%).

After conducting CSDs with patients, 23 respondents (53%) often felt confident that the patients fully understood and could make an informed decision. Twenty-seven of the respondents (63%) often felt that the patients were satisfied with the information provided during the discussion (Table 1).

When discussing the resuscitative measures with the patients, most trainees would describe details regarding cardiac arrest (74%, *N* = 32), defibrillation and cardioversion (60%, *N* = 26), chest compressions (95%, *N* = 41), and intubation with mechanical ventilation (95%, *N* = 41). The CPR complication most often discussed by trainees is breaking ribs (63%, *N* = 27), but only a minority discuss other complications such as internal bleeding (7%, *N* = 3).

During the CSDs, most trainees nearly always identify a patient's Health Care Power of Attorney (HCPOA) or surrogate, assured the patients that the code status decision can be reverted at any time, and that choosing do not resuscitate would not change the quality of care. Conversely, most trainees answered they only occasionally or almost never offer a recommendation to the patients about the code status or describe the likelihood of the patient being discharged home after a cardiac arrest.

Of the postsurvey respondents, the majority were male (64%, *N* = 18). Twelve trainees (43%) were PGY1, 3 (11%) were PGY2, 11 (39%) were PGY3, and 2 (7%) answered other. The majority were IM categorical residents (57%, *N* = 16).

Thirteen (46%) of the postsurvey respondents answered feeling extremely comfortable conducting CSDs, 12 (43%) answered feeling somewhat comfortable, and 3 (11%) answered neither comfortable nor uncomfortable (Table 1). The response option answered with a higher frequency is “extremely comfortable” and the median is “somewhat comfortable.”

After conducting CSDs, half of the respondents (50%) often felt confident that the patients fully understood and could make an informed decision, and that the patients were satisfied with the information provided (Table 1). Most of the respondents (79%, *N* = 22) almost never used the video to supplement their discussions with patients during their rotation. Eighteen (67%) respondents neither agree nor disagree that the CPR video was useful and most of the respondents (63%, *N* = 12) did watch the video themselves and used the information to guide or frame their discussions. Only two trainees (11%) showed it to the patients and only one (4%) occasionally used the Spanish version.

**FIG. 1.**
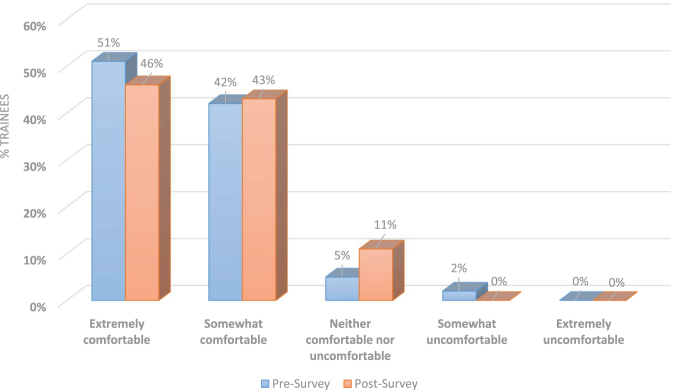
How comfortable are you discussing code status with a patient on your own?

There was no apparent difference between the pre- and postsurvey responses in terms of the residents' comfort level when conducting CSDs (Fig. 1) and their perceived patient's satisfaction and understanding of the information provided. There was no noticeable difference between the pre- and postsurvey responses in terms of specific topics covered during the conversations, including potential complications of CPR, identification of HCPOA, code status order reversibility, and recommendations offered about code status and prognosis. In the subgroup analysis of PGY1, preliminary trainees, and categorical trainees, there was no difference between the pre- and postsurvey responses.

## Discussion

Our findings demonstrated that the use of the CPR video as an interventional tool for trainee–patient CSDs neither contributed to the trainees' comfort level when conducting these discussions nor impacted their confidence in the patient's understanding and satisfaction with the CSD. CPR videos, as decision aids, are generally accepted by patients and families.^11^ Therefore, these videos should be shown to patients and families in our ICU, due to their well-established benefits.^8,11,12^

Our cohort of ICU residents, however, did not seem to be impacted by the video when it was provided to them to supplement their CSDs. Our results may have been influenced by the small sample size and the residents' low levels of engagement with the video. Perhaps using the CPR video was impractical when considering an ICU resident physician's extremely demanding role and busy schedule, particularly amid the COVID-19 pandemic.

Interestingly, most of the trainees who answered the presurvey already felt highly comfortable conducting CSDs and often felt confident with the results of these discussions. Therefore, it is plausible there is a response bias, meaning that with the low level of participation, those who volunteered to answer the surveys were those who, *a priori*, had an interest in or felt comfortable conducting CSDs.

Moreover, these findings could have been attributed to the presence of our hospital's palliative care team and their collaboration with our ICU staff, thereby making the CPR video of limited added value. Our results may also be, largely, because the clinical acumen necessary to have an effective CSD is often a skill that is cultivated over years of practice. Nevertheless, these surveys captured residents' self-reported appreciation of their performance conducting CSDs, which, in its own right, can be viewed as a project limitation.

Dickson et al.^13^ suggested that physicians' self-reported competency and comfort may not correlate with the ratings received from patients and families. Furthermore, self-reported data, in general, may not represent an accurate assessment or recollection.^4,13^ Therefore, a study involving an attending physician directly observing residents while performing CSDs and incorporating patients' feedback may be more objective.^7^ In this way, the impact of the CPR video on residents' self-reported comfort level when conducting CSDs could be directly measured against an attending's observations, and patients' reported understanding and satisfaction.

Other studies could be performed to analyze further the residents' comfort level while conducting CSDs, and gauge whether the results are related to the subject matter, confidence, and competency while engaging in the discussions.^4^

A study limitation is the inability to match the pre- and postsurveys to respondents. In turn, we are unable to explore any differences in the subset of trainees who answered both surveys. Whereas the method of administering and collecting surveys was developed to protect residents' identities during the process. This introduces a limitation in light of our methodology. Therefore, a randomized controlled trial (RCT) with two study groups is needed to establish direct comparison between the residents who use the CPR video and the control. An RCT in which subjects receive specific instructions on showing the CPR video to the patients while performing CSDs is needed to study the research hypothesis further.

Another limitation is the timing of the intervention, which started in February and ended in October. This timeframe could have introduced bias and an imbalance between the residents rotating in the ICU at the beginning versus the end of the academic year. It is plausible that rotation timing impacted residents' experiences with CSDs and, therefore, their perceived comfort. Finally, this is a single-institution study, limited to the medical ICU, limiting our findings' generalizability.

## Conclusion

In this small cohort, resident physicians did not report a change in their comfort level when conducting CSDs after the CPR video was provided to supplement these discussions. The CPR video did not change their reported confidence about the patients' understanding and satisfaction with the information provided. Our results could be related to the low practicality of the video, the timing, and the methods for data collection. Therefore, further studies, like an RCT as already specified, are needed to assess the real impact of this video on trainee–patient CSDs in the ICU.

## Abbreviations Used

CPR: cardiopulmonary resuscitation
CSDs: code status discussions
HCPOA: Health Care Power of Attorney
ICU: intensive care unit
IM: internal medicine
RCT: randomized controlled trial
